# Measurement of lower limb alignment: there are within-person differences between weight-bearing and non-weight-bearing measurement modalities

**DOI:** 10.1007/s00167-017-4636-1

**Published:** 2017-07-18

**Authors:** Daphne A. L. Schoenmakers, Peter Z. Feczko, Bert Boonen, Martijn G. M. Schotanus, Nanne P. Kort, Pieter J. Emans

**Affiliations:** 1 Department of Orthopaedic Surgery, Maastricht University Medical Center, PO Box 5800, 6202 AZ Maastricht, The Netherlands; 2 Department of Orthopaedic Surgery, Zuyderland Medical Center, Sittard, The Netherlands

**Keywords:** Total knee arthroplasty, Frontal plane limb alignment, Limb mechanical axis, Navigation, Magnetic resonance imaging, Full-leg radiographs

## Abstract

**Purpose:**

Previous studies have compared weight-bearing mechanical leg axis (MLA) measurements to non-weight-bearing measurement modalities. Most of these studies compared mean or median values and did not analyse within-person differences between measurements. This study evaluates the within-person agreement of MLA measurements between weight-bearing full-length radiographs (FLR) and non-weight-bearing measurement modalities (computer-assisted surgery (CAS) navigation or MRI).

**Materials and methods:**

Two independent observers measured the MLA on pre- and postoperative weight-bearing FLR in 168 patients. These measurements were compared to non-weight-bearing measurements obtained by CAS navigation or MRI. Absolute differences in individual subjects were calculated to determine the agreement between measurement modalities. Linear regression was used to evaluate the possibility that other independent variables impact the differences in measurements.

**Results:**

A difference was found in preoperative measurements between FLR and CAS navigation (mean of 2.5° with limit of agreement (1.96 SD) of 6.4°), as well as between FLR and MRI measurements (mean of 2.4° with limit of agreement (1.96 SD) of 6.9°). Postoperatively, the mean difference between MLA measured on FLR compared to CAS navigation was 1.5° (limit of agreement (1.96 SD) of 4.6°). Linear regression analysis showed that weight-bearing MLA measurements vary significantly from non-weight-bearing MLA measurements. Differences were more severe in patients with mediolateral instability (*p* = 0.010), age (*p* = 0.049) and ≥3° varus or valgus alignment (*p* = 0.008).

**Conclusion:**

The clinical importance of this study lies in the finding that there are within-person differences between weight-bearing and non-weight-bearing measurement modalities. This has implications for preoperative planning, performing total knee arthroplasty (TKA), and clinical follow-up after TKA surgery using CAS navigation or patient-specific instrumentation.

**Level of evidence:**

III.

## Introduction

 Accurate lower limb alignment in total knee arthroplasty (TKA) is important to improve clinical results and prosthesis survival [8, 18, 20]. Full-length weight-bearing anteroposterior radiographs (FLR) are regarded as the gold standard for determining knee joint alignment [19].

Other modalities that measure mechanical leg axis (MLA) include intra-operative computer navigation in computer-assisted surgery (CAS) and magnetic resonance imaging (MRI) in patient-specific instrumentation (PSI). Several studies have found differences between these measurement modalities [1, 6, 11, 13, 24, 28, 30–32]. However, most of these studies compared mean or median values of the measurement modalities and did not analyse within-person measurement differences. Comparing different measurements within individuals might be of greater value, as this shows the agreement between measurement modalities themselves. Neither correlation coefficients nor regression analysis are appropriate in the analysis of measurement method comparison data [2].

The discrepancy between measurement modalities may arise from a real difference in alignment between supine and weight-bearing status of the patient [5, 29]. In addition to weight-bearing conditions, previous literature has been inconsistent in which variables influence the differences between measurement modalities [7, 17, 26, 30, 32].

In this study, the authors evaluate the within-person agreement in MLA between weight-bearing measurements (FLR) and non-weight-bearing measurements (CAS navigation or MRI). In addition, independent variables that may contribute to measurement differences across modalities are examined.

## Materials and methods

This dual-centre matched cohort study was performed in two neighbouring hospitals located in the same geographical area in the Netherlands (Maastricht University Medical Center (A) and Zuyderland Medical Center (B)). A total cohort of 168 patients were analysed.

Approval of the Zuyderland Institutional Review Board was obtained for this study (16-N-66).

### Study group

Patients operated for total knee arthroplasty, who were able to undergo weight-bearing FLR, were included.

The CAS cohort consisted of 84 patients. All of whom had undergone TKA surgery by two experienced knee surgeons (PF and PE) at centre A, between 2010 and 2013). These patients were matched on age and gender to 84 patients from a consecutive cohort (*n* = 200) who were operated from 2009 to 2011 with PSI by one experienced knee surgeon (NK) in hospital B. The first 10 patients operated with PSI were excluded from matching, as they were considered to potentially influence the outcomes due to the surgeon’s learning curve. Therefore, the total cohort consisted of 168 patients. Demographic data were comparable in both groups (Table 1).

**Table 1:** Demographic data

Characteristic	Values CAS group (*n* = 84) Mean ± SD (range) or *n* (%)	Values PSI group (*n* = 84) Mean ± SD (range) or *n* (%)
Gender		
Male	47 (56%)	47 (56%)
Female	37 (44%)	37 (44%)
Age	65.8 ± 8.1 (42.6–79.3)	64.3 ± 7.3 (48.3–77.5)
Side		
Right	52 (61.9%)	52 (61.9%)
Left	32 (38.1%)	32 (38.1%)
Weight (kg)	84.6 ± 13.9 (55–119)	87.9 ± 13.3 (63–116)
Height (cm)	171.3 ± 8.1 (155–190)	171.8 ± 8.8 (150–189)
Body mass index (BMI) (kg/m^2^)	28.7 ± 3.3 (20.6–34.8)	29.9 ± 4.5 (21.8–45.0)

From five patients in the CAS group, the preoperative navigation measurements were not documented. Moreover, the proximal part of the preoperative FLR of one patient in the PSI group was missing; thus, the MLA could not be measured. From one patient in the CAS group, the postoperative CAS navigation measurements were not documented due to an intra-operative malfunctioning of the CAS navigation software. Therefore, in total six patients were excluded from preoperative analysis and one patient from the postoperative analysis.

### Imaging technique

Operations with CAS navigation were performed with an identical surgical technique using a Stryker knee navigation system (Stryker Precision Knee Navigation Software, Stryker Corp. Kalamazoo, Michigan USA). According to the manufacturer’s protocol, specific landmarks of the lower limb were digitized using a navigation pointer, with which the preoperative MLA was measured (non-weight bearing). After implanting the definitive prosthesis components, the postoperative MLA was measured again (non-weight bearing).

Before PSI surgery, all patients underwent an MRI scan of the lower limb following the protocol of the manufacturer. This MRI scan was used to create personalized positioning guides for aligning the TKA. The preoperative MLA was measured with software (Signature Personalized Patient Care Biomet, Warsaw, IN, USA) on non-weight-bearing MRI scan.

### Radiographic analysis

All patients underwent weight-bearing FLR preoperatively. These measurements were then compared to the preoperative measurements obtained by either CAS navigation or MRI. In the CAS group, postoperative FLR (12 weeks postoperatively) were also compared to the CAS navigation measurements after insertion of the total knee prosthesis. Absolute differences between measurement modalities were calculated and analysed.

For FLR, protocols were identical in both centres. All patients were bare-footed and instructed to stand upright with heels and toes touching the ground. Lower limbs were fully extended and the patella directed anteriorly. A digital ruler was projected onto the images, and three radiographs were taken. These individual radiographs were automatically merged using the digital ruler. MLA was determined using the method described by Moreland et al. [22], which is the angle formed by the intersection of a line from the centre of the femoral head to the centre of the knee and a second line from the centre of the knee to the centre of the ankle. On postoperative FLR, the centres of the femoral and tibial prosthesis components were used instead of the bony landmarks of the knee.

Measurements in the CAS group were determined in whole numbers with the iSite Enterprise software (Philips Healthcare, Foster City, California, USA). In the PSI group, measurements were determined to within 0.1° with Pacs software (Siemens Healthcare, Munich, Federal Republic of Germany) and rounded to the nearest whole number.

In order to ensure the reliability of the FLR measurements, all FLR were analysed by two independent observers in each group (DS and PF in the CAS group, and DS and BB in the PSI group). The observers were blinded for each other’s measurements as well as the measurements performed with CAS navigation or MRI. For intra-observer reliability analysis, the same researcher measured 10 pre- and 10 postoperative FLR in the CAS group and 10 preoperative FLR in the PSI group. This was done six weeks after the initial measurements were taken.

### Statistical analysis

Statistical analysis was performed using SPSS software (SPSS 21 Inc., Chicago, IL, USA).

Intra- and inter-observer reliability of radiographic MLA measurements was determined by intra-class correlation coefficients (ICCs) using a two-way random effects model for an absolute agreement definition.

To determine the agreement between measurement modalities (FLR and CAS navigation or MRI), absolute differences in individual persons were evaluated. The absolute differences between the two modalities were plotted against the average of these two measurements. The limits of agreement were used to measure the agreement between the variables and estimate the range in which 95% of the differences lie [2].

Linear regression was used to evaluate independent variables (degree of alignment deformity, body mass index (BMI), mediolateral stability during physical examination, gender, and age) that could potentially affect the differences in measurements. Statistical significance was set at *p* ≤ 0.05.

## Results

### Inter- and intra-observer reliability of radiographic measurements

All measurements of MLA on FLR demonstrated high precision with ICCs for both intra- and inter-observer reliability within a range of 0.942 and 0.989.

### Agreement between measurement modalities

**Fig. 1:**
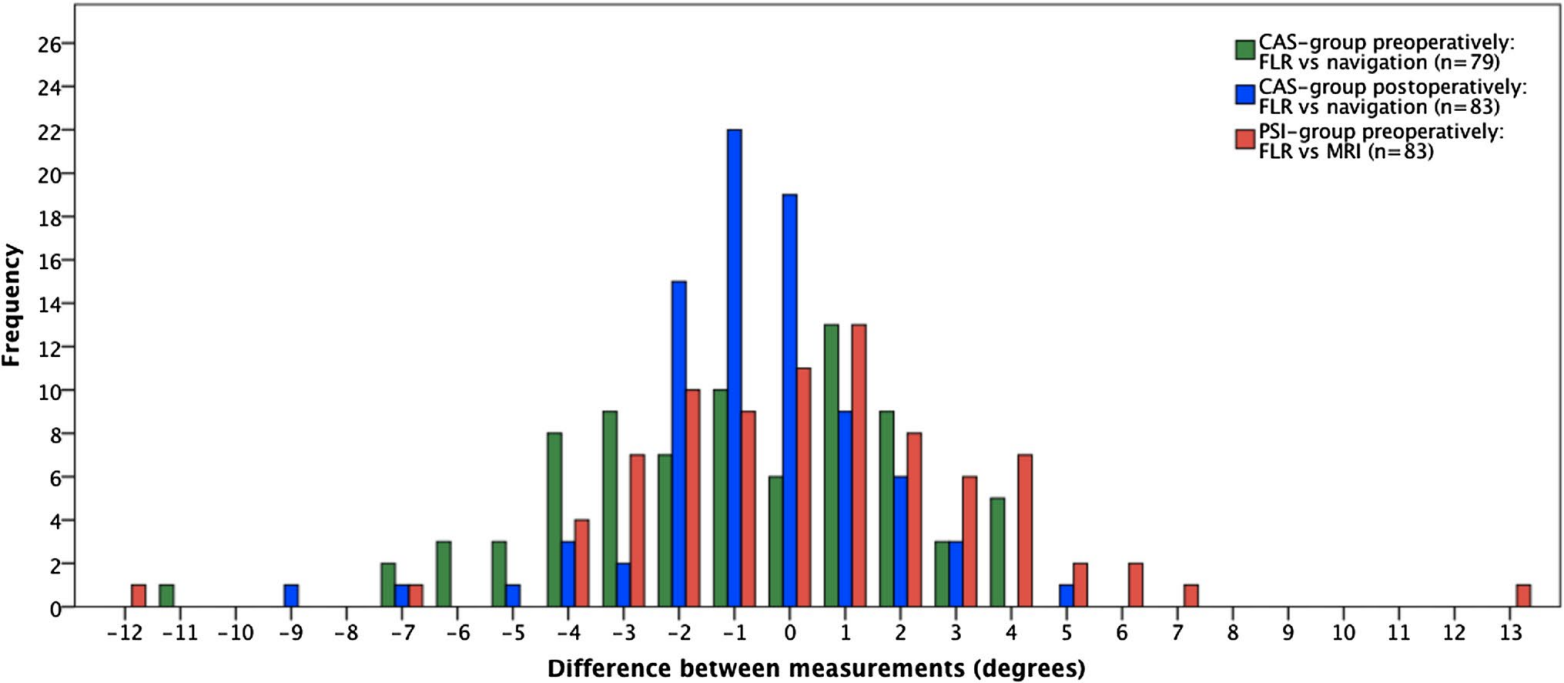
Frequency of differences in individual measurements of MLA for different measurement modalities. Difference represents the value measured on FLR minus the value measured by CAS navigation or MRI. *MLA* mechanical leg axis, *CAS* computer-assisted surgery, *PSI *patient specific instrumentation, *FLR* full-length radiograph, *MRI* magnetic resonance imaging

MLA measured on FLR versus measurements by CAS navigation showed differences >3° in 27.9% of the patients preoperatively and in 8.4% of patients postoperatively. MLA on preoperative FLR compared to preoperative measurements obtained by MRI showed differences >3° in 22.9% of the persons. There was a difference of ≥5° in nine patients in the CAS group and in eight patients in the PSI group. In five patients from the total cohort, a difference between 7° and 13° was observed (Fig. 1).

**Fig. 2:**
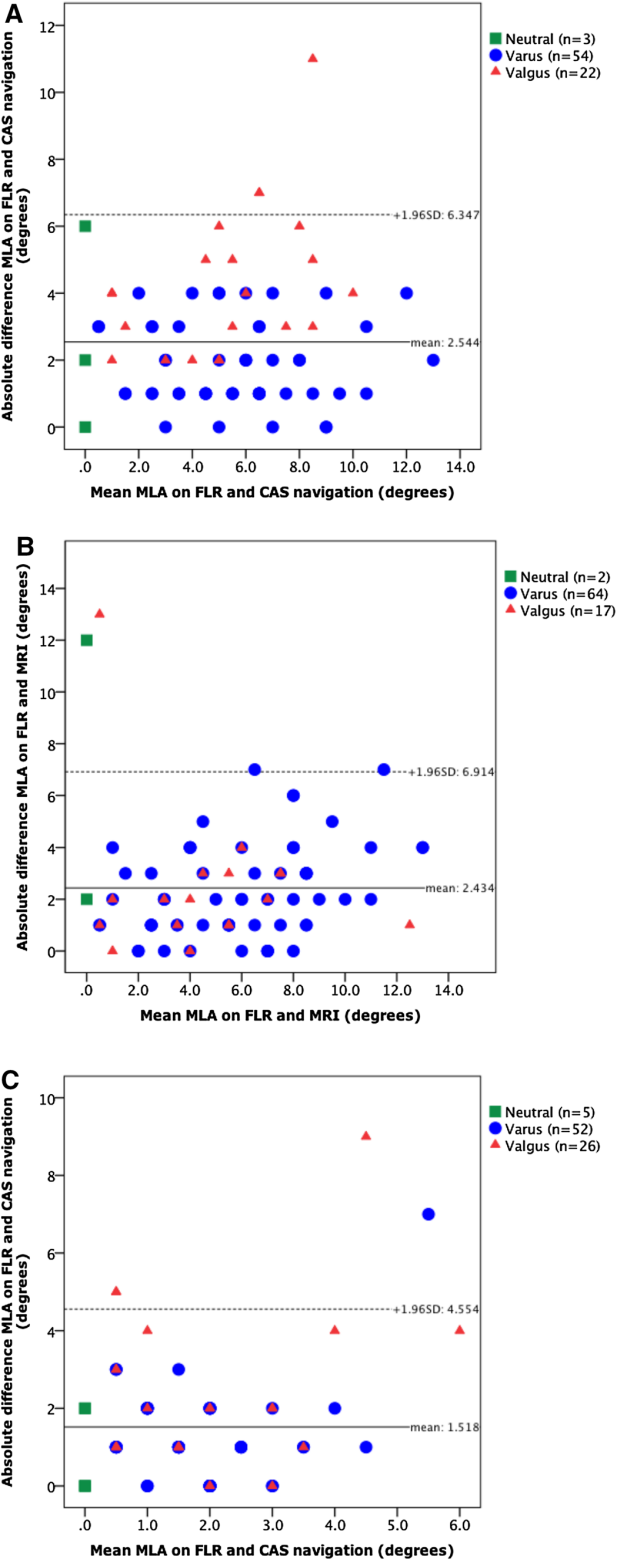
Plots with agreement of MLA measurements. Solid black lines give the mean difference in measurements and the dotted lines give the limit of agreement (mean difference ±1.96 × SD of the differences). *MLA* mechanical leg axis, *FLR* full-length radiograph, *CAS* computer-assisted surgery, *MRI* magnetic resonance imaging. **a** MLA on FLR versus CAS navigation preoperatively. **b** MLA on FLR versus MRI preoperatively. **c** MLA on FLR versus CAS navigation post-operatively

When analysing the plots based on the Bland–Altman method [2], one can observe that CAS navigation and MRI measurements differ from FLR with mean values of 2.5° and 2.4°, respectively. Postoperatively, the mean difference between MLA measured on FLR compared to CAS navigation was 1.5°. When comparing FLR with CAS navigation or MRI, the limits of agreement (1.96 SD) showed values of up to 6.4° and 6.9°, respectively, for preoperative values and 4.6° for postoperative comparison of FLR to CAS navigation (Fig. 2).

### Factors influencing differences in measurement of MLA

Multiple linear regression analysis revealed low coefficients of determination in the CAS group (*R* ^2^ of 0.132 preoperatively and 0.122 postoperatively). Differences between measurements on FLR and CAS navigation were significantly higher in preoperative measurements when mediolateral instability (during physical examination) was present (*p* = 0.010) as well as with increasing age (*p* = 0.049). For postoperative measurements, differences became significantly higher when the MLA deviated ≥3° from neutral MLA (*p* = 0.008).

Regression analysis showed no significant differences (n.s.) between measurements of FLR and MRI for any of the independent variables entered in the analysis.

## Discussion

The most important finding of the present study was that within-person MLA measurements were found to be different when comparing weight-bearing FLR to non-weight-bearing measurement modalities (CAS navigation or MRI).

This study shows high ICC, which is in line with previous literature (intra-observer reliability ICC range 0.91–1.00 [4, 30], inter-observer reliability ICC range 0.72–0.99 [4, 30]).

The discrepancy between measurement modalities may arise from a real difference in alignment between supine and the weight-bearing status of the patient, which has been recognized in prior research. Weight-bearing radiographs can differ up to 2.0° from radiographs in supine position [5, 29]. Willcox et al. [30] assessed the agreement between FLR and CAS navigation measurements of MLA and showed wider limits of agreement of −9.4° and 8.6° preoperatively and −5.0° to 5.4° postoperatively. This is line with the findings of the current work.

Winter et al. [31] assessed the relationship between preoperative FLR and MRI measurements of MLA and showed a correlation between the two techniques (Pearson’s correlation coefficient (*r*) = 0.88) and a large absolute variability in measurements in the same patient, with differences up to 8°. As previously noticed, correlation does not equate to agreement [2]. Based on the absolute differences described by Winter et al. [31], a mean difference of 2.6° could be calculated from their data, which is similar to the mean difference found in the present study (2.4°). Paternostre et al. [24] also assessed the differences in MLA measurements between FLR and MRI and found no significant difference (evaluation by Student’s t test). They found differences >3° in 23% of patients, which was similar to the current study (differences higher than 3° in 22.9% of the persons). Moreover, they stated that the difference seems to be related to higher Kellgren–Lawrence stages where deformity increased under load-bearing conditions.

In the present study, it was found that discrepancies were higher in preoperative measurements compared to postoperative measurements. Other authors have also concluded that preoperative measurements involve a higher degree of ligamentous imbalance, which may lead to greater alignment deformity while weight-bearing [23]. Knees are balanced after TKA, and therefore, the postoperative difference between weight-bearing and non-weight-bearing gets smaller [23].

In five patients, an outlying difference between 7° and 13° was observed. This could be the result of several factors such as fixed flexion deformity, incorrect placement or loosening of navigation trackers, ligamentous imbalance, and measurement or administration errors. The complete analysis was repeated without these five outliers. The results from this analysis did not differ from our previous results including outliers. Only the mean difference decreased marginally within a range of 0.1°–0.3°, as expected.

It has been noted in previous literature that the risk of inaccuracy of MLA is more likely in the presence of flexion of the knee or rotation of the leg [17, 26]. Measurements of MLA with CAS navigation or with MRI are independent from rotation or flexion since they are three-dimensional. Previous studies demonstrated that CAS navigation measurements are precise [12, 33]. The system-determined error has been described within 1° in the coronal plane [10, 25, 33]. Nonetheless, there is a potential for error since the registration process of CAS navigation is subject to inter- or intra-surgeon variations when demarcating correct landmark registration, or potential loosening of the tracker [27, 33]. For PSI, measurements are also subject to movements of the patient during scanning in MRI.

Previous literature is non-concurrent on indicating independent factors that influence MLA measurements [6, 7, 24, 28, 30–32]. In addition to weight-bearing status, variable factors may influence the measurements resulting in increased discrepancy. Our findings show that mediolateral instability and age had a significant influence on the differences between preoperative FLR and CAS navigation measurements. A ≥3° alignment deformity from neutral MLA resulted in significantly higher differences in postoperative CAS group measurements. However, these differences were only of very small clinical relevance with *R* ^2^ ranging from 0.122 to 0.284.

The present study contains some limitations. The aforementioned potential errors were not investigated in either the CAS navigation’s registration process or the MRI scan. A further shortcoming of this study is the lacking determination of flexion and rotation data. As a result, they could not be analysed as confounding variables. Another limitation of the present study results from the fact that two patient samples were used. Obtaining all three modalities (FLR, CAS navigation, and MRI) in the same patient sample would be desirable to reduce bias. Finally, other measurement modalities (e.g. computed tomography (CT), single-photon emission computed tomography (SPECT/CT) or 3D reconstructions using SterEOS software) have been evaluated in previous literature [3, 9, 14–16, 21], but were not included in the present study. Comparison of more measurement modalities in individual persons might be of added value in future research.

## Conclusion

The clinical importance of this study lies in the finding that differences were observed in within-person MLA measurements. A mean difference of up to 2.5° between weight-bearing and non-weight-bearing MLA measurements has implications for preoperative planning, performing TKA, and clinical follow-up after TKA surgery using CAS navigation or PSI.
